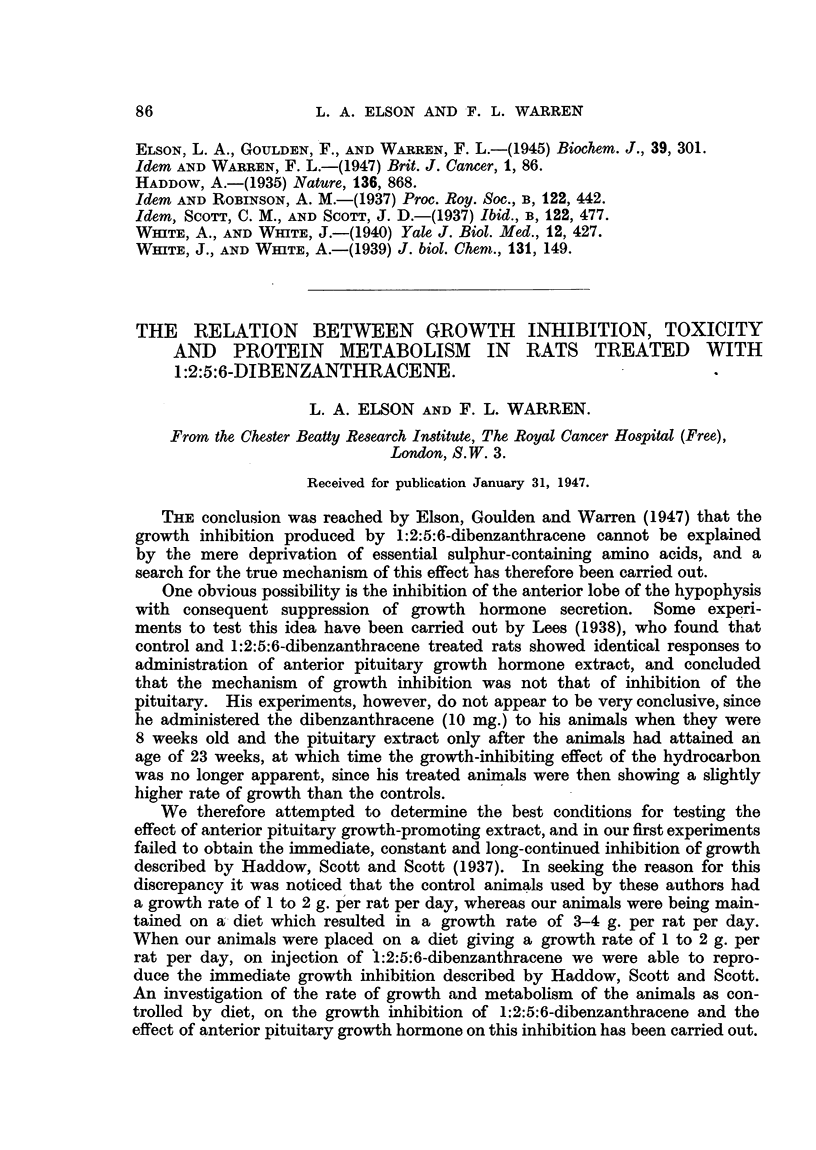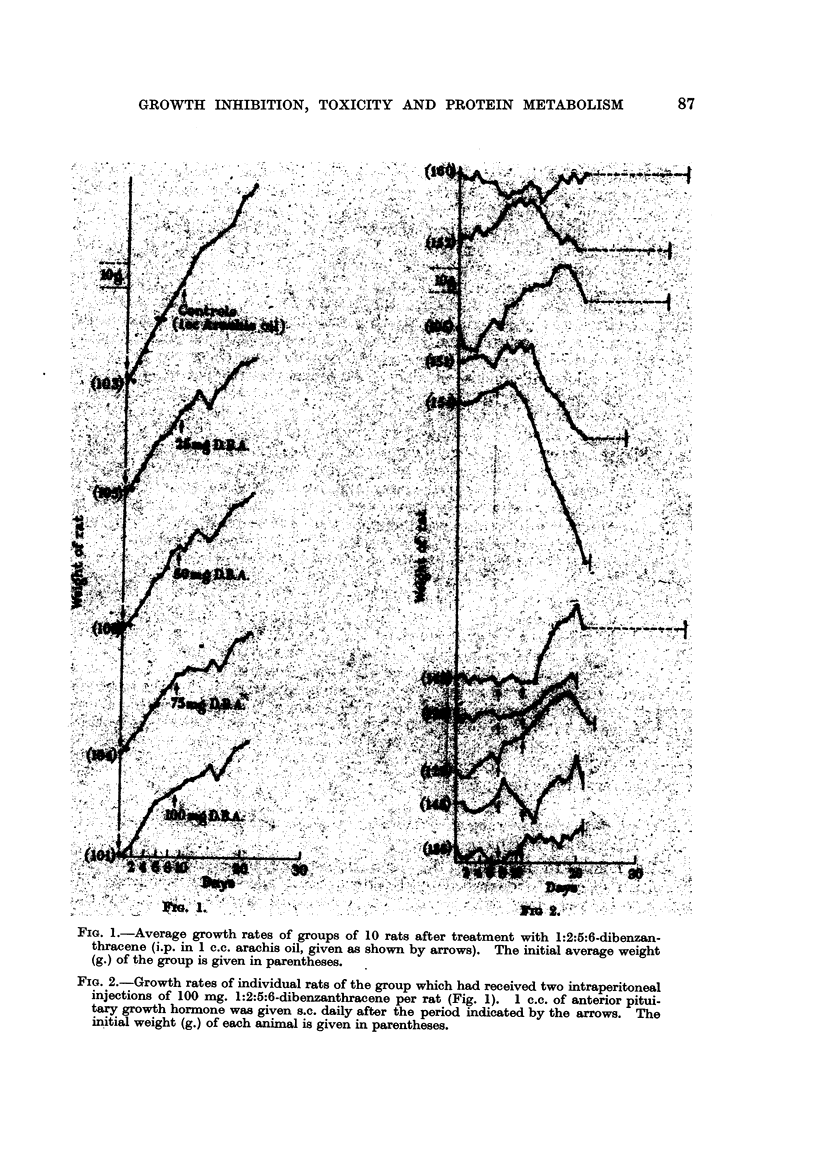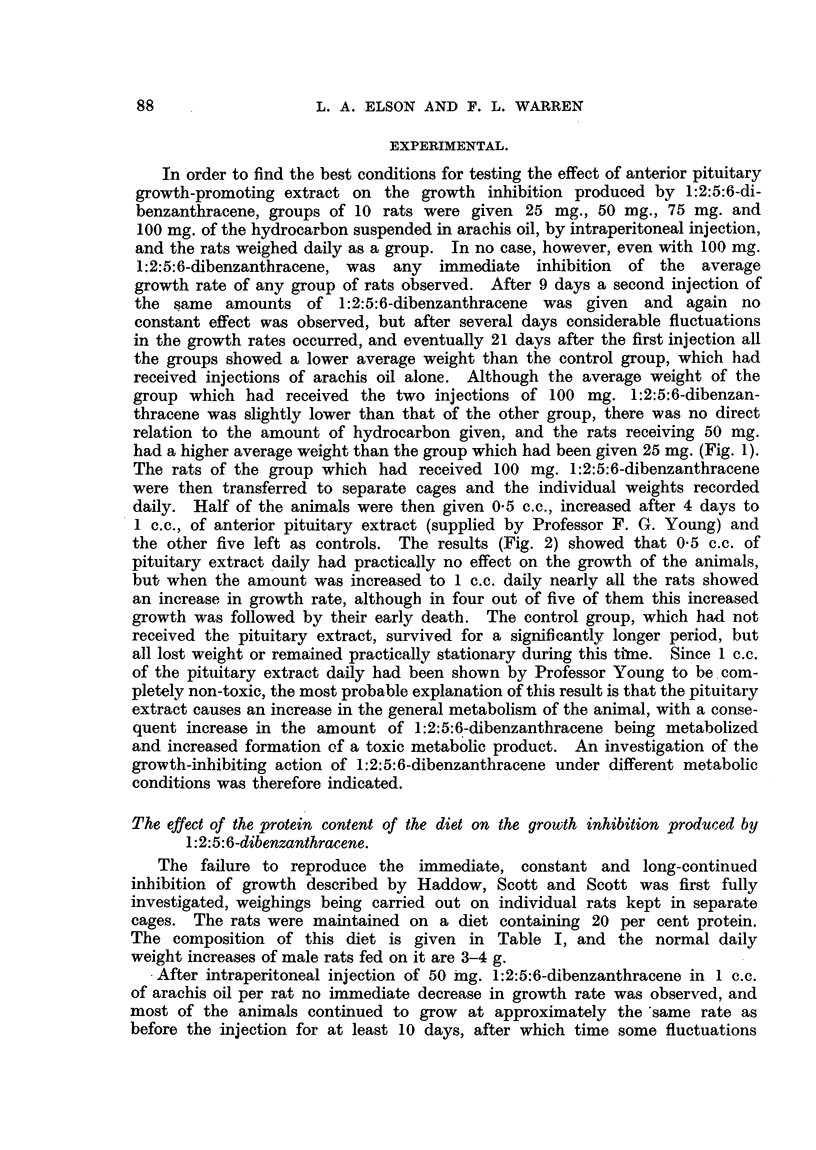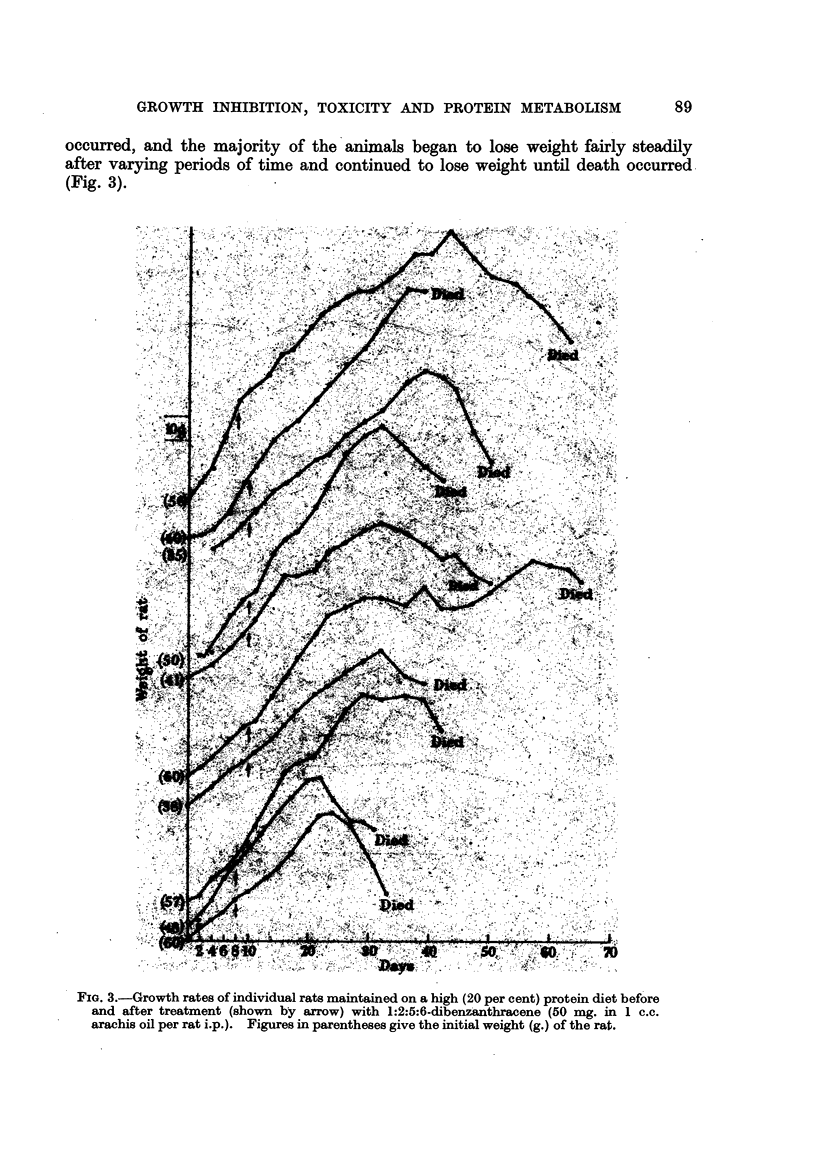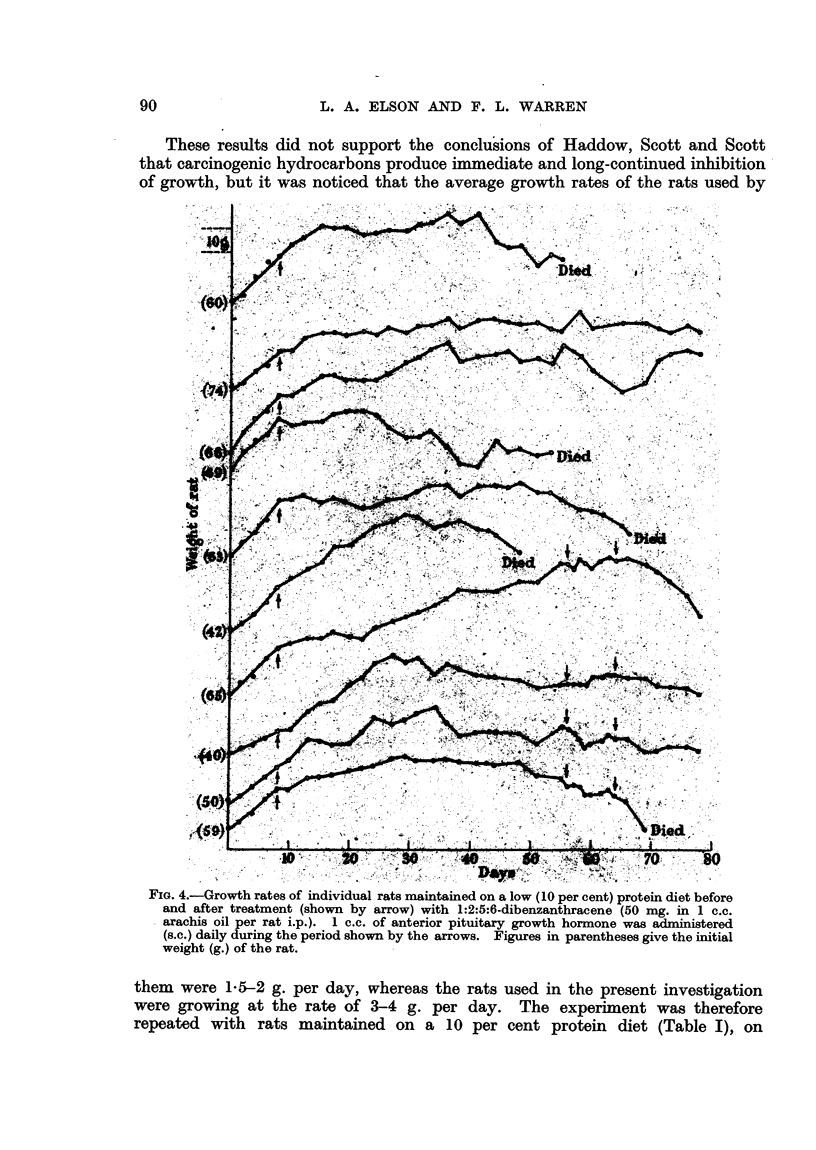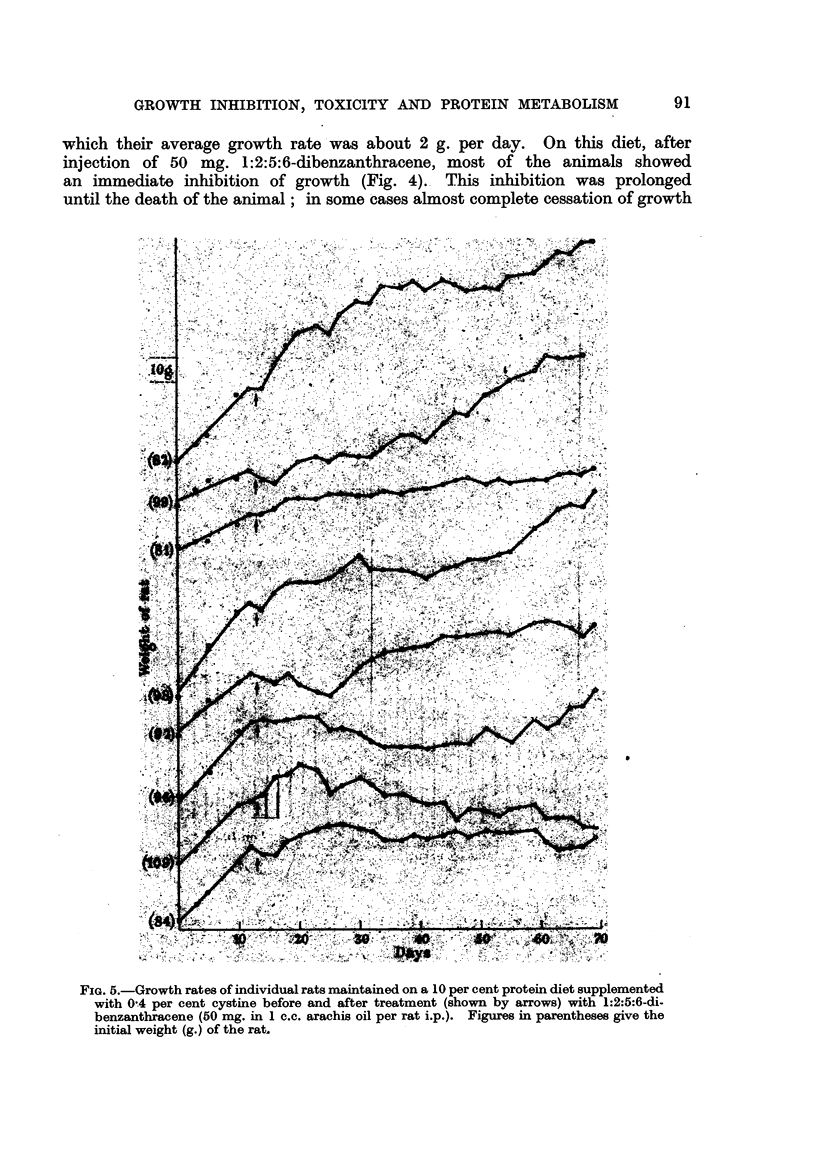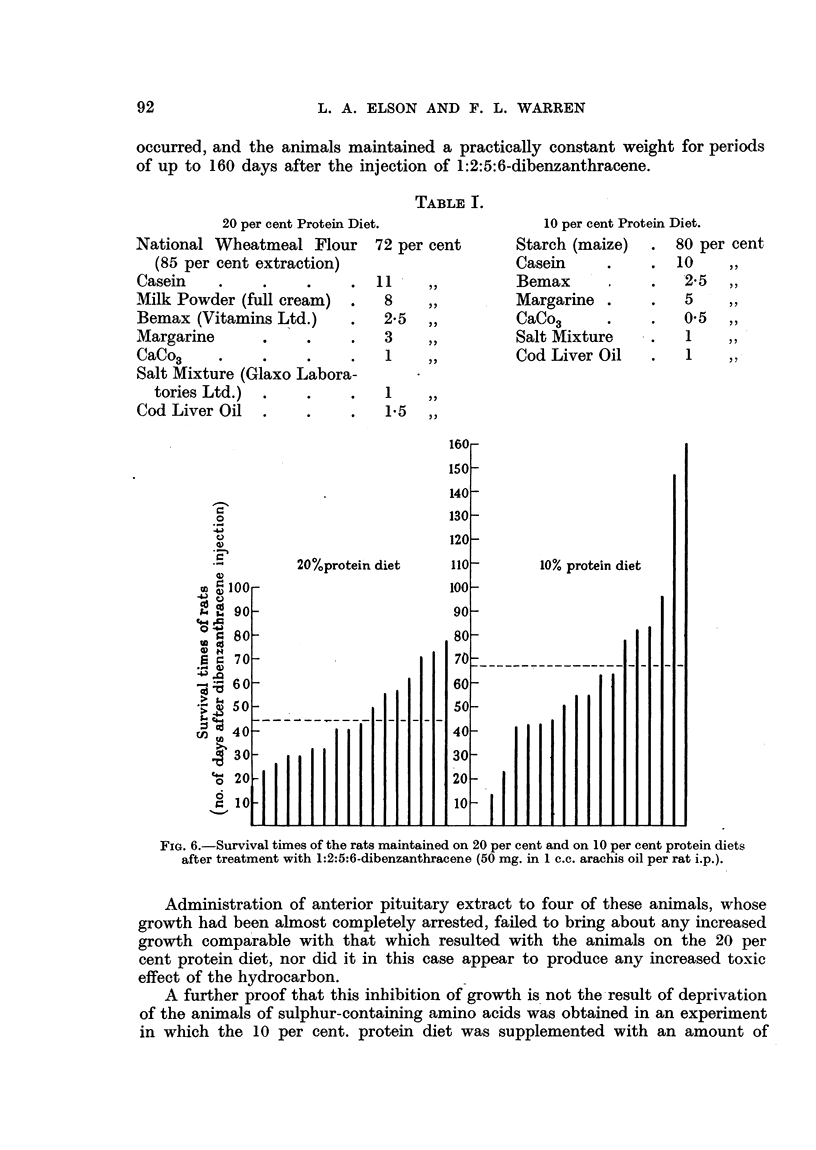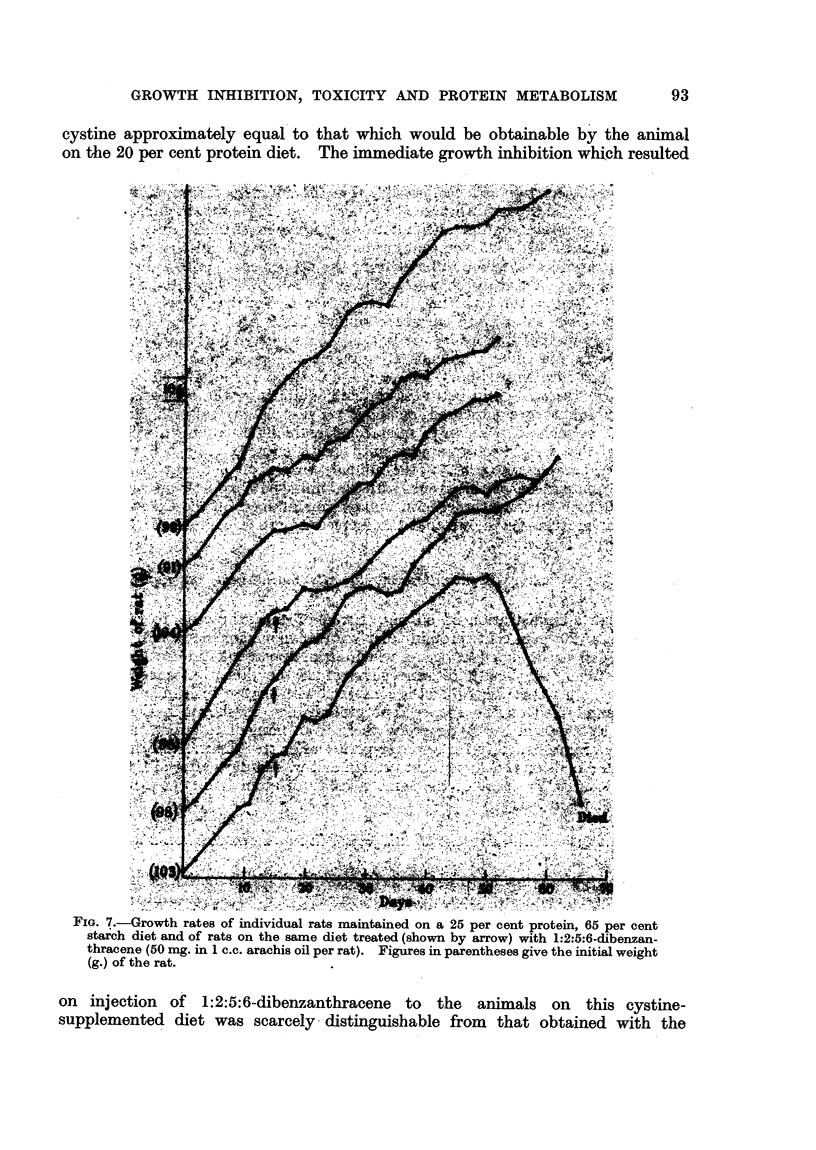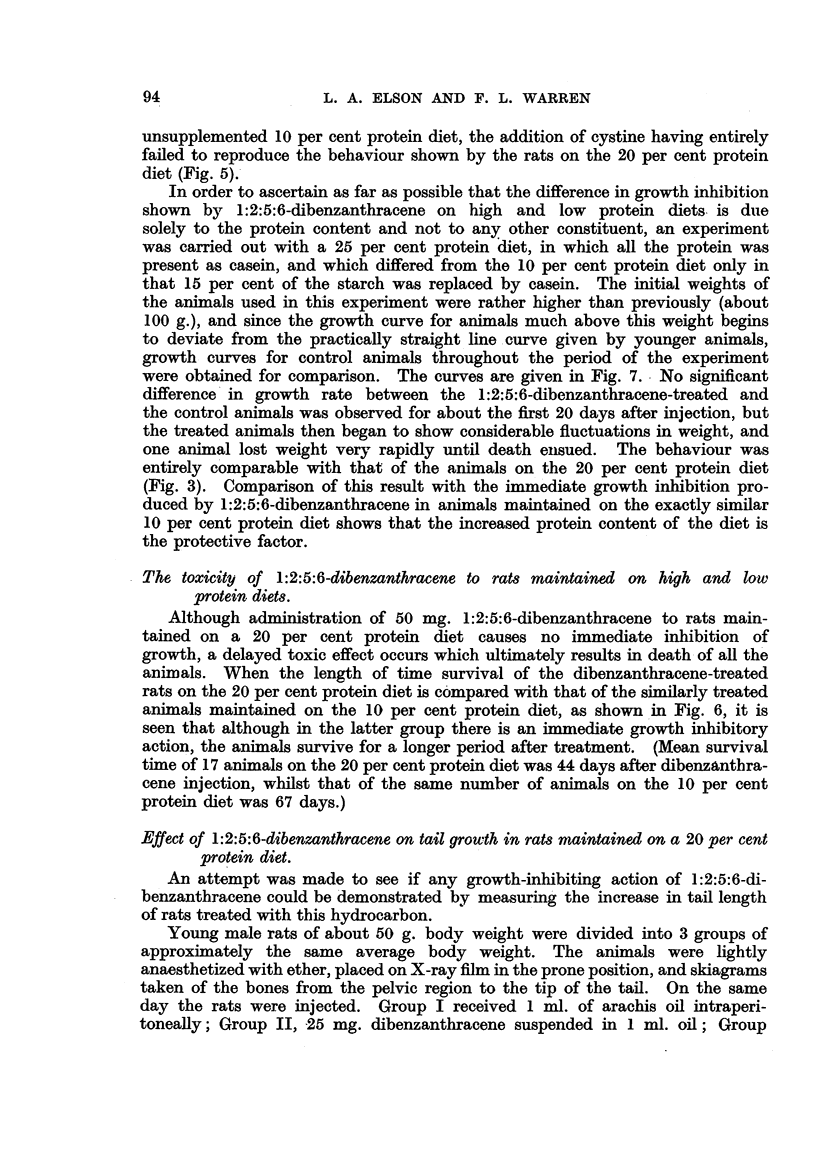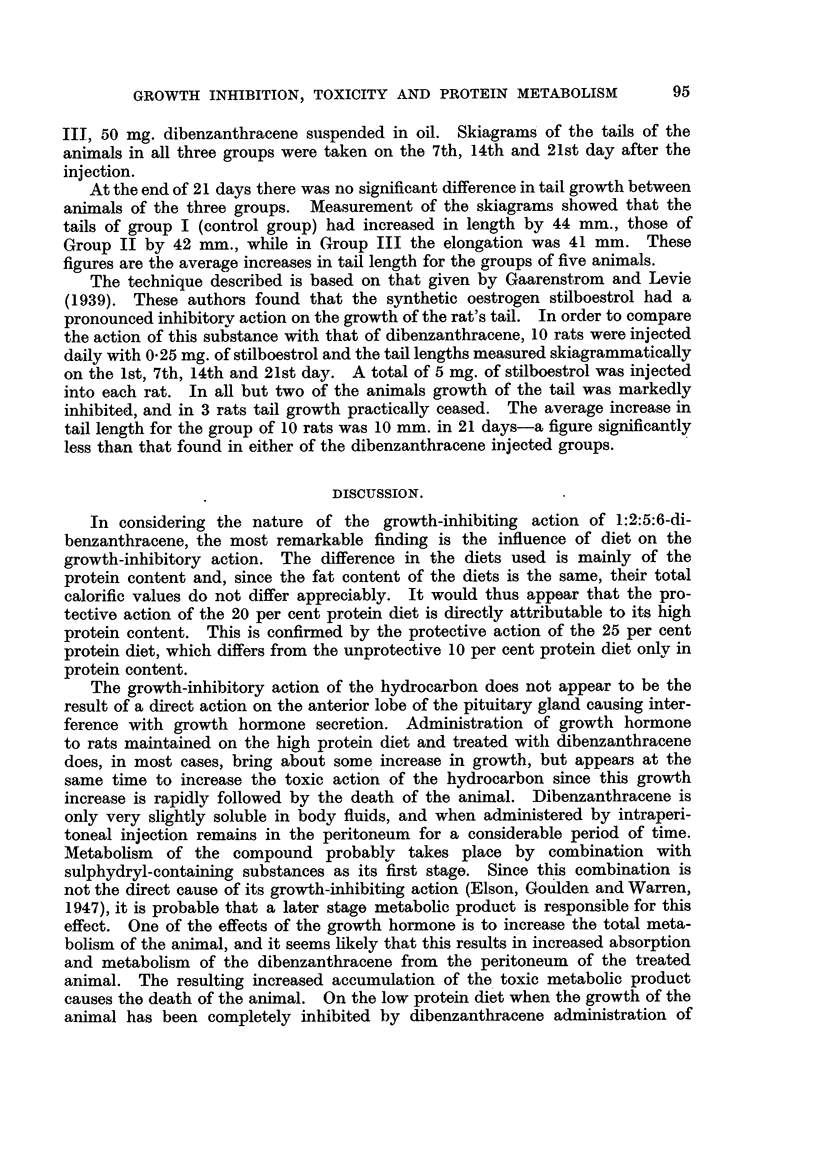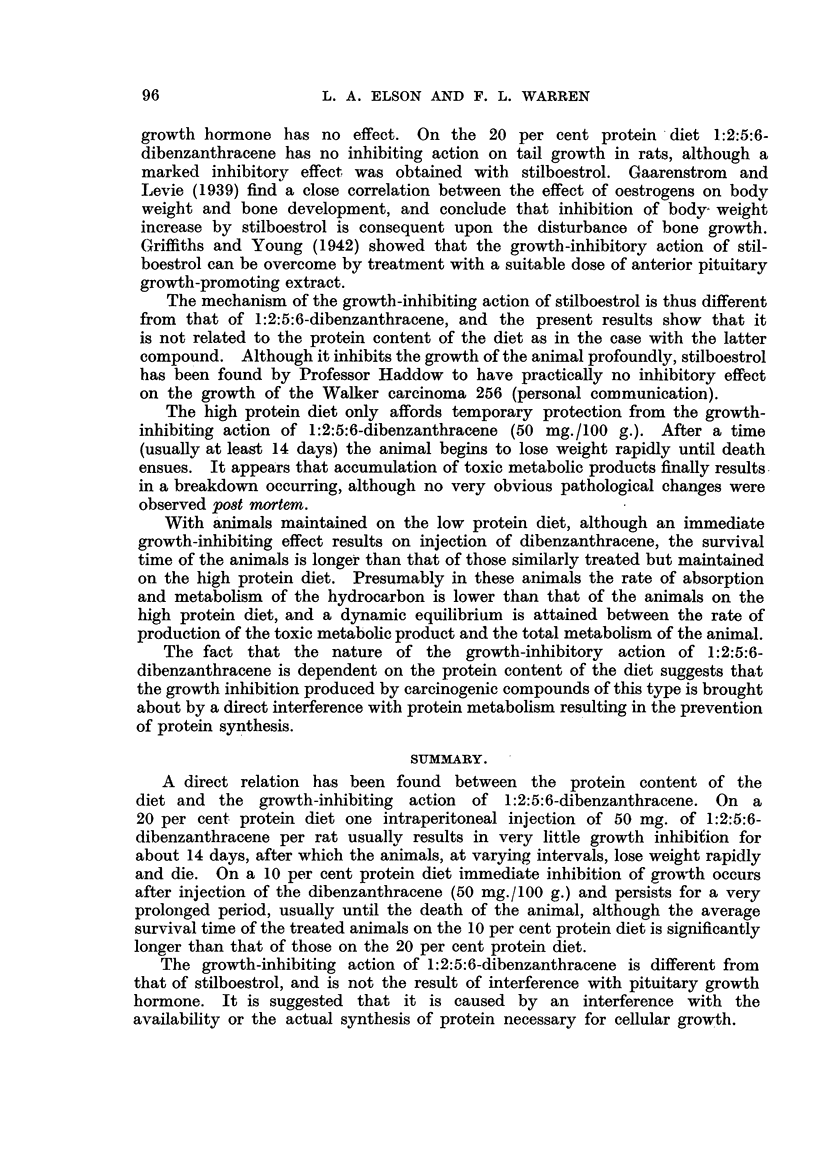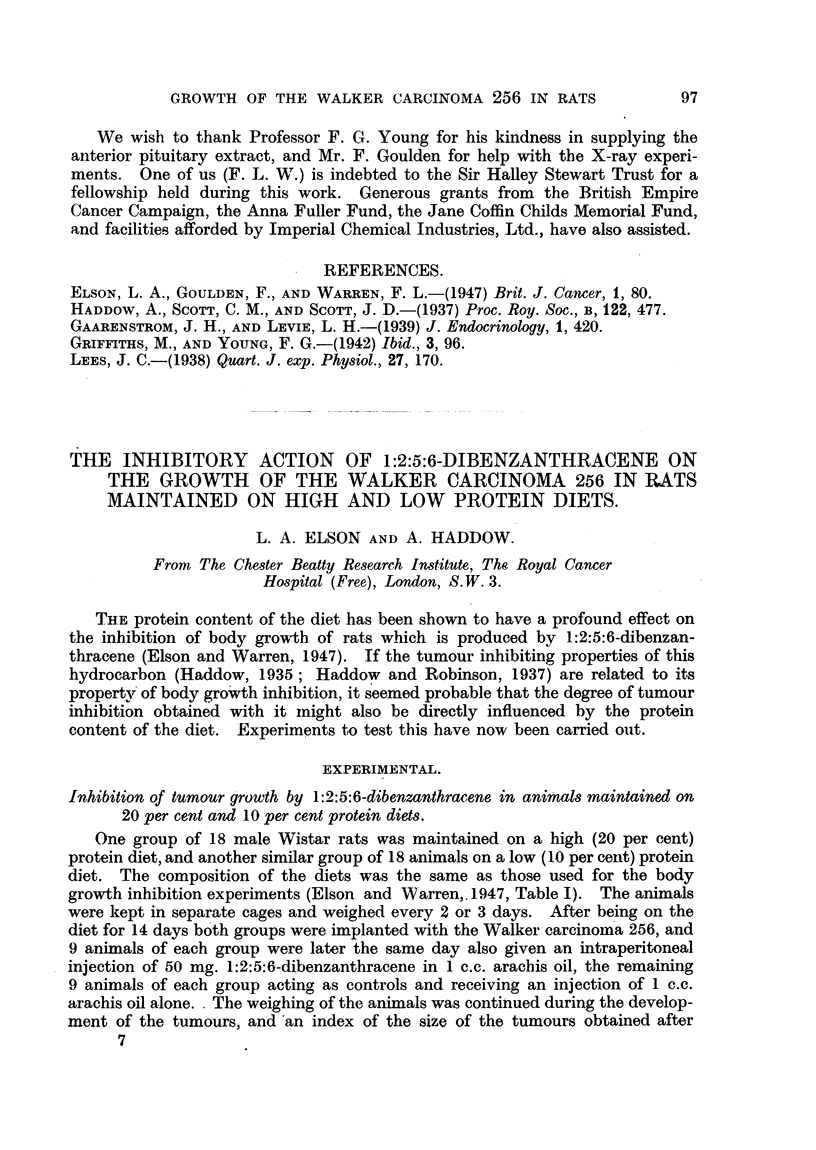# The Relation between Growth Inhibition, Toxicity and Protein Metabolism in Rats Treated with 1:2:5:6-Dibenzanthracene

**DOI:** 10.1038/bjc.1947.11

**Published:** 1947-03

**Authors:** L. A. Elson, F. L. Warren


### THE RELATION BETWEEN GROWTH INHIBITION, TOXICITY AND PROTEIN METABOLISM IN RATS TREATED WITH 1:2:5:6-DIBENZANTHRACENE.

L. A. ELSON AND F. L. WARREN.

*From the Chester Beatty Research Institute, The Royal Cancer Hospital (Free), London, S.W. 3.*

Received for publication January 31, 1947.

THE conclusion was reached by Elson, Goulden and Warren (1947) that the growth inhibition produced by 1:2:5:6-dibenzanthracene cannot be explained by the mere deprivation of essential sulphur-containing amino acids, and a search for the true mechanism of this effect has therefore been carried out.

One obvious possibility is the inhibition of the anterior lobe of the hypophysis with consequent suppression of growth hormone secretion. Some experiments to test this idea have been carried out by Lees (1938), who found that control and 1:2:5:6-dibenzanthracene treated rats showed identical responses to administration of anterior pituitary growth hormone extract, and concluded that the mechanism of growth inhibition was not that of inhibition of the pituitary. His experiments, however, do not appear to be very conclusive, since he administered the dibenzanthracene (10 mg.) to his animals when they were 8 weeks old and the pituitary extract only after the animals had attained an age of 23 weeks, at which time the growth-inhibiting effect of the hydrocarbon was no longer apparent, since his treated animals were then showing a slightly higher rate of growth than the controls.

We therefore attempted to determine the best conditions for testing the effect of anterior pituitary growth-promoting extract, and in our first experiments failed to obtain the immediate, constant and long-continued inhibition of growth described by Haddow, Scott and Scott (1937). In seeking the reason for this discrepancy it was noticed that the control animals used by these authors had a growth rate of 1 to 2 g. per rat per day, whereas our animals were being maintained on a diet which resulted in a growth rate of 3–4 g. per rat per day. When our animals were placed on a diet giving a growth rate of 1 to 2 g. per rat per day, on injection of 1:2:5:6-dibenzanthracene we were able to reproduce the immediate growth inhibition described by Haddow, Scott and Scott. An investigation of the rate of growth and metabolism of the animals as controlled by diet, on the growth inhibition of 1:2:5:6-dibenzanthracene and the effect of anterior pituitary growth hormone on this inhibition has been carried out.

FIG. 1.

FIG 2.

FIG. 1.—Average growth rates of groups of 10 rats after treatment with 1:2:5:6-dibenzanthracene (i.p. in 1 c.c. arachis oil, given as shown by arrows). The initial average weight (g.) of the group is given in parentheses.

FIG. 2.—Growth rates of individual rats of the group which had received two intraperitoneal injections of 100 mg. 1:2:5:6-dibenzanthracene per rat (Fig. 1). 1 c.c. of anterior pituitary growth hormone was given s.c. daily after the period indicated by the arrows. The initial weight (g.) of each animal is given in parentheses.

### EXPERIMENTAL.

In order to find the best conditions for testing the effect of anterior pituitary growth-promoting extract on the growth inhibition produced by 1:2:5:6-dibenzanthracene, groups of 10 rats were given 25 mg., 50 mg., 75 mg. and 100 mg. of the hydrocarbon suspended in arachis oil, by intraperitoneal injection, and the rats weighed daily as a group. In no case, however, even with 100 mg. 1:2:5:6-dibenzanthracene, was any immediate inhibition of the average growth rate of any group of rats observed. After 9 days a second injection of the same amounts of 1:2:5:6-dibenzanthracene was given and again no constant effect was observed, but after several days considerable fluctuations in the growth rates occurred, and eventually 21 days after the first injection all the groups showed a lower average weight than the control group, which had received injections of arachis oil alone. Although the average weight of the group which had received the two injections of 100 mg. 1:2:5:6-dibenzanthracene was slightly lower than that of the other group, there was no direct relation to the amount of hydrocarbon given, and the rats receiving 50 mg. had a higher average weight than the group which had been given 25 mg. (Fig. 1). The rats of the group which had received 100 mg. 1:2:5:6-dibenzanthracene were then transferred to separate cages and the individual weights recorded daily. Half of the animals were then given 0·5 c.c., increased after 4 days to 1 c.c., of anterior pituitary extract (supplied by Professor F. G. Young) and the other five left as controls. The results (Fig. 2) showed that 0·5 c.c. of pituitary extract daily had practically no effect on the growth of the animals, but when the amount was increased to 1 c.c. daily nearly all the rats showed an increase in growth rate, although in four out of five of them this increased growth was followed by their early death. The control group, which had not received the pituitary extract, survived for a significantly longer period, but all lost weight or remained practically stationary during this time. Since 1 c.c. of the pituitary extract daily had been shown by Professor Young to be completely non-toxic, the most probable explanation of this result is that the pituitary extract causes an increase in the general metabolism of the animal, with a consequent increase in the amount of 1:2:5:6-dibenzanthracene being metabolized and increased formation of a toxic metabolic product. An investigation of the growth-inhibiting action of 1:2:5:6-dibenzanthracene under different metabolic conditions was therefore indicated.

### *The effect of the protein content of the diet on the growth inhibition produced by* 1:2:5:6-*dibenzanthracene.*

The failure to reproduce the immediate, constant and long-continued inhibition of growth described by Haddow, Scott and Scott was first fully investigated, weighings being carried out on individual rats kept in separate cages. The rats were maintained on a diet containing 20 per cent protein. The composition of this diet is given in Table I, and the normal daily weight increases of male rats fed on it are 3–4 g.

After intraperitoneal injection of 50 mg. 1:2:5:6-dibenzanthracene in 1 c.c. of arachis oil per rat no immediate decrease in growth rate was observed, and most of the animals continued to grow at approximately the same rate as before the injection for at least 10 days, after which time some fluctuations occurred, and the majority of the animals began to lose weight fairly steadily after varying periods of time and continued to lose weight until death occurred (Fig. 3).

FIG. 3.—Growth rates of individual rats maintained on a high (20 per cent) protein diet before and after treatment (shown by arrow) with 1:2:5:6-dibenzanthracene (50 mg. in 1 c.c. arachis oil per rat i.p.). Figures in parentheses give the initial weight (g.) of the rat.

These results did not support the conclusions of Haddow, Scott and Scott that carcinogenic hydrocarbons produce immediate and long-continued inhibition of growth, but it was noticed that the average growth rates of the rats used by

FIG. 4.—Growth rates of individual rats maintained on a low (10 per cent) protein diet before and after treatment (shown by arrow) with 1:2:5:6-dibenzanthracene (50 mg. in 1 c.c. arachis oil per rat i.p.). 1 c.c. of anterior pituitary growth hormone was administered (s.c.) daily during the period shown by the arrows. Figures in parentheses give the initial weight (g.) of the rat.

them were 1·5–2 g. per day, whereas the rats used in the present investigation were growing at the rate of 3–4 g. per day. The experiment was therefore repeated with rats maintained on a 10 per cent protein diet (Table I), on which their average growth rate was about 2 g. per day. On this diet, after injection of 50 mg. 1:2:5:6-dibenzanthracene, most of the animals showed an immediate inhibition of growth (Fig. 4). This inhibition was prolonged until the death of the animal ; in some cases almost complete cessation of growth

FIG. 5.—Growth rates of individual rats maintained on a 10 per cent protein diet supplemented with 0·4 per cent cystine before and after treatment (shown by arrows) with 1:2:5:6-dibenzanthracene (50 mg. in 1 c.c. arachis oil per rat i.p.). Figures in parentheses give the initial weight (g.) of the rat.

occurred, and the animals maintained a practically constant weight for periods of up to 160 days after the injection of 1:2:5:6-dibenzanthracene.

TABLE I.

20 per cent Protein Diet.

| | |
|---|---|
| National Wheatmeal Flour (85 per cent extraction) | 72 per cent |
| Casein . . . . . | 11 ,, |
| Milk Powder (full cream) . | 8 ,, |
| Bemax (Vitamins Ltd.) . | 2·5 ,, |
| Margarine . . . | 3 ,, |
| $CaCo_3$ . . . . . | 1 ,, |
| Salt Mixture (Glaxo Laboratories Ltd.) . . . | 1 ,, |
| Cod Liver Oil . . . | 1·5 ,, |

10 per cent Protein Diet.

| | |
|---|---|
| Starch (maize) . | 80 per cent |
| Casein . . | 10 ,, |
| Bemax . . | 2·5 ,, |
| Margarine . . | 5 ,, |
| $CaCo_3$ . . | 0·5 ,, |
| Salt Mixture . | 1 ,, |
| Cod Liver Oil . | 1 ,, |

FIG. 6.—Survival times of the rats maintained on 20 per cent and on 10 per cent protein diets after treatment with 1:2:5:6-dibenzanthracene (50 mg. in 1 c.c. arachis oil per rat i.p.).

Administration of anterior pituitary extract to four of these animals, whose growth had been almost completely arrested, failed to bring about any increased growth comparable with that which resulted with the animals on the 20 per cent protein diet, nor did it in this case appear to produce any increased toxic effect of the hydrocarbon.

A further proof that this inhibition of growth is not the result of deprivation of the animals of sulphur-containing amino acids was obtained in an experiment in which the 10 per cent. protein diet was supplemented with an amount of cystine approximately equal to that which would be obtainable by the animal on the 20 per cent protein diet. The immediate growth inhibition which resulted

FIG. 7.—Growth rates of individual rats maintained on a 25 per cent protein, 65 per cent starch diet and of rats on the same diet treated (shown by arrow) with 1:2:5:6-dibenzanthracene (50 mg. in 1 c.c. arachis oil per rat). Figures in parentheses give the initial weight (g.) of the rat.

on injection of 1:2:5:6-dibenzanthracene to the animals on this cystine-supplemented diet was scarcely distinguishable from that obtained with the unsupplemented 10 per cent protein diet, the addition of cystine having entirely failed to reproduce the behaviour shown by the rats on the 20 per cent protein diet (Fig. 5).

In order to ascertain as far as possible that the difference in growth inhibition shown by 1:2:5:6-dibenzanthracene on high and low protein diets is due solely to the protein content and not to any other constituent, an experiment was carried out with a 25 per cent protein diet, in which all the protein was present as casein, and which differed from the 10 per cent protein diet only in that 15 per cent of the starch was replaced by casein. The initial weights of the animals used in this experiment were rather higher than previously (about 100 g.), and since the growth curve for animals much above this weight begins to deviate from the practically straight line curve given by younger animals, growth curves for control animals throughout the period of the experiment were obtained for comparison. The curves are given in Fig. 7. No significant difference in growth rate between the 1:2:5:6-dibenzanthracene-treated and the control animals was observed for about the first 20 days after injection, but the treated animals then began to show considerable fluctuations in weight, and one animal lost weight very rapidly until death ensued. The behaviour was entirely comparable with that of the animals on the 20 per cent protein diet (Fig. 3). Comparison of this result with the immediate growth inhibition produced by 1:2:5:6-dibenzanthracene in animals maintained on the exactly similar 10 per cent protein diet shows that the increased protein content of the diet is the protective factor.

*The toxicity of* 1:2:5:6-*dibenzanthracene to rats maintained on high and low protein diets.*

Although administration of 50 mg. 1:2:5:6-dibenzanthracene to rats maintained on a 20 per cent protein diet causes no immediate inhibition of growth, a delayed toxic effect occurs which ultimately results in death of all the animals. When the length of time survival of the dibenzanthracene-treated rats on the 20 per cent protein diet is compared with that of the similarly treated animals maintained on the 10 per cent protein diet, as shown in Fig. 6, it is seen that although in the latter group there is an immediate growth inhibitory action, the animals survive for a longer period after treatment. (Mean survival time of 17 animals on the 20 per cent protein diet was 44 days after dibenzanthracene injection, whilst that of the same number of animals on the 10 per cent protein diet was 67 days.)

*Effect of* 1:2:5:6-*dibenzanthracene on tail growth in rats maintained on a* 20 *per cent protein diet.*

An attempt was made to see if any growth-inhibiting action of 1:2:5:6-dibenzanthracene could be demonstrated by measuring the increase in tail length of rats treated with this hydrocarbon.

Young male rats of about 50 g. body weight were divided into 3 groups of approximately the same average body weight. The animals were lightly anaesthetized with ether, placed on X-ray film in the prone position, and skiagrams taken of the bones from the pelvic region to the tip of the tail. On the same day the rats were injected. Group I received 1 ml. of arachis oil intraperitoneally; Group II, 25 mg. dibenzanthracene suspended in 1 ml. oil; Group

III, 50 mg. dibenzanthracene suspended in oil. Skiagrams of the tails of the animals in all three groups were taken on the 7th, 14th and 21st day after the injection.

At the end of 21 days there was no significant difference in tail growth between animals of the three groups. Measurement of the skiagrams showed that the tails of group I (control group) had increased in length by 44 mm., those of Group II by 42 mm., while in Group III the elongation was 41 mm. These figures are the average increases in tail length for the groups of five animals.

The technique described is based on that given by Gaarenstrom and Levie (1939). These authors found that the synthetic oestrogen stilboestrol had a pronounced inhibitory action on the growth of the rat's tail. In order to compare the action of this substance with that of dibenzanthracene, 10 rats were injected daily with 0·25 mg. of stilboestrol and the tail lengths measured skiagrammatically on the 1st, 7th, 14th and 21st day. A total of 5 mg. of stilboestrol was injected into each rat. In all but two of the animals growth of the tail was markedly inhibited, and in 3 rats tail growth practically ceased. The average increase in tail length for the group of 10 rats was 10 mm. in 21 days—a figure significantly less than that found in either of the dibenzanthracene injected groups.

### DISCUSSION.

In considering the nature of the growth-inhibiting action of 1:2:5:6-dibenzanthracene, the most remarkable finding is the influence of diet on the growth-inhibitory action. The difference in the diets used is mainly of the protein content and, since the fat content of the diets is the same, their total calorific values do not differ appreciably. It would thus appear that the protective action of the 20 per cent protein diet is directly attributable to its high protein content. This is confirmed by the protective action of the 25 per cent protein diet, which differs from the unprotective 10 per cent protein diet only in protein content.

The growth-inhibitory action of the hydrocarbon does not appear to be the result of a direct action on the anterior lobe of the pituitary gland causing interference with growth hormone secretion. Administration of growth hormone to rats maintained on the high protein diet and treated with dibenzanthracene does, in most cases, bring about some increase in growth, but appears at the same time to increase the toxic action of the hydrocarbon since this growth increase is rapidly followed by the death of the animal. Dibenzanthracene is only very slightly soluble in body fluids, and when administered by intraperitoneal injection remains in the peritoneum for a considerable period of time. Metabolism of the compound probably takes place by combination with sulphydryl-containing substances as its first stage. Since this combination is not the direct cause of its growth-inhibiting action (Elson, Goulden and Warren, 1947), it is probable that a later stage metabolic product is responsible for this effect. One of the effects of the growth hormone is to increase the total metabolism of the animal, and it seems likely that this results in increased absorption and metabolism of the dibenzanthracene from the peritoneum of the treated animal. The resulting increased accumulation of the toxic metabolic product causes the death of the animal. On the low protein diet when the growth of the animal has been completely inhibited by dibenzanthracene administration of growth hormone has no effect. On the 20 per cent protein diet 1:2:5:6-dibenzanthracene has no inhibiting action on tail growth in rats, although a marked inhibitory effect was obtained with stilboestrol. Gaarenstrom and Levie (1939) find a close correlation between the effect of oestrogens on body weight and bone development, and conclude that inhibition of body weight increase by stilboestrol is consequent upon the disturbance of bone growth. Griffiths and Young (1942) showed that the growth-inhibitory action of stilboestrol can be overcome by treatment with a suitable dose of anterior pituitary growth-promoting extract.

The mechanism of the growth-inhibiting action of stilboestrol is thus different from that of 1:2:5:6-dibenzanthracene, and the present results show that it is not related to the protein content of the diet as in the case with the latter compound. Although it inhibits the growth of the animal profoundly, stilboestrol has been found by Professor Haddow to have practically no inhibitory effect on the growth of the Walker carcinoma 256 (personal communication).

The high protein diet only affords temporary protection from the growth-inhibiting action of 1:2:5:6-dibenzanthracene (50 mg./100 g.). After a time (usually at least 14 days) the animal begins to lose weight rapidly until death ensues. It appears that accumulation of toxic metabolic products finally results in a breakdown occurring, although no very obvious pathological changes were observed *post mortem.*

With animals maintained on the low protein diet, although an immediate growth-inhibiting effect results on injection of dibenzanthracene, the survival time of the animals is longer than that of those similarly treated but maintained on the high protein diet. Presumably in these animals the rate of absorption and metabolism of the hydrocarbon is lower than that of the animals on the high protein diet, and a dynamic equilibrium is attained between the rate of production of the toxic metabolic product and the total metabolism of the animal.

The fact that the nature of the growth-inhibitory action of 1:2:5:6-dibenzanthracene is dependent on the protein content of the diet suggests that the growth inhibition produced by carcinogenic compounds of this type is brought about by a direct interference with protein metabolism resulting in the prevention of protein synthesis.

### SUMMARY.

A direct relation has been found between the protein content of the diet and the growth-inhibiting action of 1:2:5:6-dibenzanthracene. On a 20 per cent protein diet one intraperitoneal injection of 50 mg. of 1:2:5:6-dibenzanthracene per rat usually results in very little growth inhibition for about 14 days, after which the animals, at varying intervals, lose weight rapidly and die. On a 10 per cent protein diet immediate inhibition of growth occurs after injection of the dibenzanthracene (50 mg./100 g.) and persists for a very prolonged period, usually until the death of the animal, although the average survival time of the treated animals on the 10 per cent protein diet is significantly longer than that of those on the 20 per cent protein diet.

The growth-inhibiting action of 1:2:5:6-dibenzanthracene is different from that of stilboestrol, and is not the result of interference with pituitary growth hormone. It is suggested that it is caused by an interference with the availability or the actual synthesis of protein necessary for cellular growth.

We wish to thank Professor F. G. Young for his kindness in supplying the anterior pituitary extract, and Mr. F. Goulden for help with the X-ray experiments. One of us (F. L. W.) is indebted to the Sir Halley Stewart Trust for a fellowship held during this work. Generous grants from the British Empire Cancer Campaign, the Anna Fuller Fund, the Jane Coffin Childs Memorial Fund, and facilities afforded by Imperial Chemical Industries, Ltd., have also assisted.

### REFERENCES.

ELSON, L. A., GOULDEN, F., AND WARREN, F. L.—(1947) *Brit. J. Cancer*, **1**, 80.
HADDOW, A., SCOTT, C. M., AND SCOTT, J. D.—(1937) *Proc. Roy. Soc.*, B, **122**, 477.
GAARENSTROM, J. H., AND LEVIE, L. H.—(1939) *J. Endocrinology*, **1**, 420.
GRIFFITHS, M., AND YOUNG, F. G.—(1942) *Ibid.*, **3**, 96.
LEES, J. C.—(1938) *Quart. J. exp. Physiol.*, **27**, 170.

### THE INHIBITORY ACTION OF 1:2:5:6-DIBENZANTHRACENE ON THE GROWTH OF THE WALKER CARCINOMA 256 IN RATS MAINTAINED ON HIGH AND LOW PROTEIN DIETS.

L. A. ELSON AND A. HADDOW.